# Differences in the Clinical Characteristics of Male Patients With Different Ages of Rosacea: A Retrospective Study of 215 Male Outpatients

**DOI:** 10.1111/jocd.16620

**Published:** 2024-10-07

**Authors:** Yuwei Huang, Siliang Chen, Xu Liu, Dan Du, Xian Jiang

**Affiliations:** ^1^ Department of Dermatology, West China Hospital Sichuan University Chengdu China; ^2^ Laboratory of Dermatology, Clinical Institute of Inflammation and Immunology, Frontiers Science Center for Disease‐Related Molecular Network, West China Hospital Sichuan University Chengdu China

**Keywords:** age, clinical study, male, retrospective studies, rosacea

## Abstract

**Background:**

Rosacea is more common in women and Caucasians, leading to little research on rosacea in Asian men. Additionally, there is limited research on the patients across different age groups.

**Aims:**

The aim of this study is to analyze and compare the characteristics of male patients of rosacea among different age groups.

**Methods:**

A retrospective analysis was conducted on 215 male patients with rosacea, investigating their characteristics, clinical symptoms, exacerbating factors, complications, psychological status, and treatment, as well as exploring factors influencing the early onset of male rosacea.

**Results:**

The patients were divided into three age groups (≤ 30 years, 31–44 years, and ≥ 45 years), with the study revealing an average age of 38.59 ± 13.13 years among the patients. The most common subtype of rosacea in men was erythematotelangiectatic rosacea (ETR), followed by phymatous rosacea (PhR). The main reported features included persistent erythema (87.4%) and telangiectasia (71.2%), predominantly affecting the nose (58.6%) and cheeks (56.3%). Twenty‐six percent of patients reported concurrent skin diseases, with 14.0% reporting systemic diseases. Significant differences were observed among different age groups regarding family history, clinical features, lesion distribution, symptom severity, aggravating factors, presence of systemic diseases, and treatment preferences. Subjective skin typing, Fitzpatrick phototype, and positive family history were identified as factors influencing the age of onset of rosacea in men.

**Conclusion:**

Male patients with rosacea exhibit distinct clinical characteristics, with a greater prevalence of nasal involvement and nasal lesions among male patients. Clinical features vary among different age groups, with patients aged ≥ 45 experiencing more complex and severe symptoms. Patients aged ≤ 30 may be more influenced by genetic factors and have higher treatment expectations.

## 1 Introduction

Rosacea is a chronic inflammatory condition primarily affecting the central facial region [1]. Clinical manifestations in patients may include flushing, persistent erythema, papules, pustules, phymatous changes, burning, and edema, with some patients also experiencing ocular symptoms [2, 3]. The pathogenesis of rosacea has not been fully elucidated, and factors such as temperature, emotions, diet, and lifestyle changes are believed to exacerbate symptoms [4]. Rosacea is a visibly disfiguring condition that is closely linked to low self‐esteem and poor psychological well‐being in patients [5]. It is also associated with other skin conditions, such as acne [6] and perioral dermatitis [7], and systemic diseases. Its complexity and tendency for relapse pose significant challenges in clinical management, adding to the burden of disease in healthcare settings [8].

The incidence of rosacea is estimated to be between 5% and 10% [9, 10, 11], with fair‐skinned individuals (Fitzpatrick skin phototypes I and II) being more susceptible than those with skin of color (Fitzpatrick skin phototypes III–VI) [9]. In the Asian population, the incidence of rosacea is approximately 3% [12]. Sexwise, rosacea is known to predominantly affect women aged 20–50 years, with its prevalence increasing with age [13]. A meta‐analysis comprising 140 458 rosacea patients revealed that 71% were female, with the majority being approximately 50 years old [14]. Male patients with rosacea account for approximately 3.9% of the population [13]. Studies have suggested that male patients with rosacea may present with more severe papulopustular lesions and a greater incidence of nasal rosacea [15, 16, 17, 18]. However, there is currently a lack of research specifically focusing on the clinical characteristics of male patients with rosacea, especially among Asians.

Understanding the disease presentation of male patients from different ethnic backgrounds is beneficial for the clinical diagnosis and treatment of rosacea, particularly since the incidence of rosacea is greater in Caucasians. Additionally, the clinical features of rosacea may vary with age due to differences in testosterone levels and skin conditions among males of different ages. To comprehensively understand rosacea and tailor specific treatment approaches for different patients, we conducted a retrospective analysis of 215 male outpatients diagnosed with rosacea, with a focus on elucidating and comparing clinical characteristics across different ages, as well as exploring factors influencing the age of onset of male rosacea.

## 2 Method

### 2.1 Patients

Retrospectively, male patients who were diagnosed with rosacea by dermatologists during outpatient treatment from December 1, 2019, to December 1, 2024, were included based on the diagnostic criteria outlined in the 2019 Global ROSacea COnsus (ROSCO) [9]. This study was approved by the Medical Ethics Committee of the West China Hospital of Sichuan University (Clinical Trials.gov ID: 2024–237). Informed consent was obtained from all patients. The exclusion criteria included individuals with flushing or persistent facial erythema not definitively diagnosed with rosacea and those unable to comprehend the questionnaire content or who were able to collaborate with the researchers. When grouping by age, considering sample balance and intergroup stability, patients were divided into three age groups based on their age at the time of being diagnosed with rosacea in our outpatient department: Group One (≤ 30 years old), Group Two (31–44 years old), and Group Three (≥ 45 years old). Patients were divided into groups based on the age at which they first reported the onset of rosacea: onset age ≤ 30 years group and onset age > 30 years group.

### 2.2 Data Collected

Dermatologists or trained researchers utilized standardized questionnaires to collect information and assess cutaneous rosacea features, severity, and the psychological state of enrolled patients. The collected information included patient demographics, family history, phenotypes, lesion distribution, rosacea subtypes, aggravating factors, comorbidities, and treatment preferences. The evaluation of rosacea subtypes and phenotype severity used the NRS Expert Committee's published rosacea standard grading system [19] to score the severity of each phenotype, providing a global assessment on the same scale (ranging from 0 to 3, where 0: absent; 1: mild; 2: moderate; 3: severe). Persistent erythema and telangiectasia were diagnosed with the assistance of VISIA and dermoscopy.

Based on the previous literature and clinical experience, eleven potential aggravating factors for rosacea have been identified, including temperature increase, temperature reduction, dry environment, humid environment, smoking, alcohol consumption, spicy food consumption, exercise, sun exposure, the application of cosmetics, the use or changing of skincare products, and emotional changes. These factors can be directly or indirectly altered in a patient's life.

Psychological state assessment utilized the hospital anxiety and depression scale (HADS) developed by Zigmond and Snaith in 1983 [20]. The scale consists of 14 items (scored from 0 to 3) and two sets of test questions that assess the states of anxiety and depression. Scores of 0–7 indicate normalcy, 8–10 indicate mild anxiety and depression, 11–14 indicate moderate anxiety and depression, and 15–21 indicate severe anxiety and depression. This scale is widely used for the rapid assessment of patient anxiety and depression.

### 2.3 Statistical Analyses

The statistical analyses were conducted using SPSS statistical software (version 28; IBM). Quantitative variables are presented as the mean values ± standard deviations, whereas categorical data are reported as frequencies and percentages. Categorical variables were evaluated using Fisher's exact test or the χ^2^ test. One‐way ANOVA was used to analyze quantitative variables among three or more groups, followed by the homogeneity of variance test. Nonnormally distributed data were compared using the Kruskal–Wallis test. Odds ratios (ORs) and 95% CIs were calculated to assess the potential risk factors for the onset of rosacea in individuals aged ≤ 30 years using logistic regression models. A two‐sided *p* value < 0.05 was considered to indicate statistical significance.

## 3 Results

### 3.1 Patient Characteristics and Rosacea Features

Between December 1, 2019, and December 1, 2024, 1830 patients received a diagnosis of rosacea during outpatient consultations. Among them, 215 (11.7%) were male. The clinical data of 215 male patients with rosacea were retrospectively collected and categorized into three groups: 69 (32.1%) patients were aged ≤ 30 years, 77 (35.8%) patients were aged 31–44 years, and 69 (32.1%) patients were aged ≥ 45 years. The mean age of the included patients was 38.59 ± 13.13 years, the mean age at onset was 32.68 ± 13.21 years, and the mean duration of the disease was 5.86 ± 6.12 years, with the ≥ 45‐year‐old group being the oldest (8.09 ± 8.61), with significant group differences (*p* = 0.013). The patient characteristics and rosacea features of the different age groups are summarized in Table 1.

**TABLE 1:** Clinical characteristics and rosacea features of the different age groups [*n* (%), mean ± SD].

Variable	Total (*n* = 215)	≤ 30 (*n* = 69)	31–44 (*n* = 77)	≥ 45 (*n* = 69)	*p*
Age	38.59 ± 13.13	24.90 ± 3.16	36.44 ± 4.11	54.67 ± 7.32	**< 0.001**
Age of onset	32.68 ± 13.21	19.99 ± 3.69	31.79 ± 6.49	46.36 ± 11.56	**< 0.001**
Disease duration	5.86 ± 6.12	5.00 ± 3.85	4.68 ± 4.58	8.09 ± 8.61	**0.013**
**Subjective skin typing**					
Dry	33 (15.3)	7 (10.1)	12 (15.6)	14 (20.3)	0.275
Normal	35 (16.3)	7 (10.1)	15 (19.5)	13 (18.8)	0.259
Oily	68 (31.6)	28 (40.6)	21 (27.3)	19 (27.5)	0.159
Combination	29 (13.5)	14 (20.3)	10 (13.0)	5 (7.2)	0.086
Sensitive	50 (23.3)	13 (18.8)	19 (24.7)	18 (26.1)	0.540
**Fitzpatrick phototypes**					
II	6 (2.8)	5 (7.2)	1 (1.3)	0 (0.0)	**0.029**
III	185 (86.0)	63 (91.3)	66 (85.7)	56 (81.2)	0.232
IV	24 (11.2)	1 (1.4)	10 (13.0)	13 (18.8)	**0.004**
Family history	22 (10.2)	12 (17.4)	7 (9.1)	3 (4,3)	**0.034**
**Cutaneous rosacea feature**					
Flushing	126 (55.8)	37 (53.6)	51 (66.2)	38 (55.1)	0.234
Persistent facial erythema	188 (87.4)	58 (84.1)	67 (87.0)	63 (91.3)	0.422
Papules or pustules	132 (61.4)	40 (58.0)	44 (57.1)	48 (69.6)	0.242
Telangiectasia	153 (71.2)	45 (65.2)	54 (70.1)	54 (78.3)	0.238
Phymatous changes	91 (42.3)	33 (47.8)	23 (29.9)	35 (50.7)	**0.021**
Burning/stinging sensation	144 (67.0)	46 (66.7)	56 (72.7)	42 (60.9)	0.314
Dry sensation	105 (48.8)	33 (47.8)	35 (45.5)	37 (53.6)	0.632
Edema	54 (25.1)	16 (23.2)	21 (27.3)	17 (24.6)	0.886
Pruritus	88 (40.9)	24 (34.8)	27 (35.1)	37 (53.6)	**0.036**
**Lesion distribution**					
Cheeks	121 (56.3)	27 (39.1)	46 (59.7)	48 (69.6)	**0.001**
Nose	126 (58.6)	52 (75.4)	35 (45.5)	39 (56.5)	**0.001**
Perioral area	27 (12.6)	10 (14.5)	12 (15.6)	5 (7.2)	0.267
Forehead	31 (14.4)	9 (13.0)	13 (16.9)	9 (13.0)	0.805
Eyes	8 (3.7)	2 (2.9)	2 (2.6)	4 (5.8)	0.663
**Rosacea subtypes**					
ETR	65 (30.2)	18 (26.1)	29 (37.7)	18 (26.1)	0.205
PPR	26 (12.1)	12 (17.4)	6 (7.8)	8 (11.6)	0.219
PhR	41 (19.1)	15 (21.7)	14 (18.2)	12 (17.4)	0.821
ETR + PPR	54 (25.1)	13 (18.8)	23 (29.9)	18 (26.1)	0.315
ETR + PhR	6 (2.8)	2 (2.9)	2 (2.6)	2 (2.9)	1.000
PPR + ETR	10 (4.7)	5 (7.5)	3 (3.9)	2 (2.9)	0.470
ETR + PPR + PhR	13 (6.0)	4 (5.8)	0 (0.0)	9 (13.0)	**0.001**

*Note:* Statistically significant *p* values are in bold.

Abbreviations: ETR, erythematotelangiectatic rosacea; PhR, phymatous rosacea; PPR, papulopustular rosacea.

A family history of rosacea was reported by 10.2% of the patients, with the highest prevalence observed in the group aged ≤ 30 years (17.4%). The difference in family history incidence between the age groups was statistically significant (*p* = 0.034). Regarding rosacea phenotypes, persistent facial erythema (87.4%) and telangiectasia (71.2%) were the most common manifestations. A total of 42.3% of patients experienced phymatous changes, which varied significantly between age groups (*p* = 0.021). The ≥ 45‐year‐old age group reported a significantly greater incidence of pruritus (53.6%) than did the other two groups (*p* = 0.036).

Lesions were predominantly distributed on the nose (58.6%) and cheeks (56.3%) of male patients. Notably, lesions were more commonly found on the nose (75.4%) in patients younger than 30 years (*p* = 0.001), whereas in patients older than 45 years, the cheeks were the most prevalent site (69.6%) for lesion distribution (*p* = 0.001). Among the single subtypes, the majority of patients had erythematotelangiectatic rosacea (ETR), followed by phymatous rosacea (PhR) and papulopustular rosacea (PPR). Among the mixed subtypes, the most common was ETR + PPR.

### 3.2 Severity and Aggravating Factors

The average severity grades of rosacea were significantly different among the different age groups for flushing (*p* = 0.01), persistent erythema (*p* = 0.001), papules or pustules (*p* = 0.02), telangiectasia (*p* = 0.035), and phymatous changes (*p* = 0.006). Specifically, group two exhibited more severe flushing (1.04 ± 0.20), whereas the remaining four demonstrated higher mean severity scores for group three.

Among the 11 identified factors aggravating rosacea, a majority of patients (58.1%) reported that temperature increases and spicy foods (57.2%) triggered their symptoms. Additionally, alcohol (47.0%), sun exposure (42.8%), and emotional changes (38.6%) were also commonly cited as aggravating factors. Upon conducting between‐group comparisons, temperature reduction (*p* = 0.027), humidity (*p* = 0.039), exercise (*p* < 0.001), use or change of skincare products (*p* = 0.008), and emotional changes (*p* = 0.002) revealed significant differences. These results are summarized in Table 2.

**TABLE 2:** Rosacea severity and aggravating factors of the different age groups [*n* (%), mean ± SD].

Variable	Total (*n* = 215)	≤ 30 (*n* = 69)	31–44 (*n* = 77)	≥ 45 (*n* = 69)	*p*
Severity					
Severity of flushing	0.88 ± 0.86	0.65 ± 0.68	1.04 ± 0.90	0.93 ± 0.93	**0.01**
Severity of persistent erythema	1.30 ± 0.81	1.04 ± 0.67	1.30 ± 0.83	1.55 ± 0.83	**0.001**
Severity of papules or pustules	0.92 ± 0.93	0.78 ± 0.84	0.81 ± 0.87	1.17 ± 1.04	**0.02**
Severity of telangiectasia	0.95 ± 0.76	0.78 ± 0.68	0.96 ± 0.79	1.12 ± 0.78	**0.035**
Severity of phymatous changes	0.59 ± 0.77	0.59 ± 0.69	0.39 ± 0.65	0.81 ± 0.91	**0.006**
Severity of edema	0.34 ± 0.65	0.29 ± 0.57	0.34 ± 0.60	0.39 ± 0.77	0.658
Severity of burning sensation	0.84 ± 0.78	0.75 ± 0.74	0.96 ± 0.77	0.78 ± 0.82	0.214
Severity of stinging sensation	0.50 ± 0.70	0.45 ± 0.68	0.55 ± 0.68	0.51 ± 0.74	0.707
Severity of dry sensation	0.73 ± 0.88	0.62 ± 0.79	0.73 ± 0.93	0.86 ± 0.91	0.302
Aggravating factors					
Temperature increment	125 (58.1)	37 (53.6)	49 (63.6)	39 (56.5)	0.449
Temperature reduction	21 (9.8)	12 (15.9)	6 (7.8)	3 (4.3)	**0.027**
Dry environments	33 (15.3)	8 (11.6)	17 (22.1)	8 (11.6)	0.123
Humid environments	9 (4.2)	5 (7.2)	0 (0.0)	4 (5.8)	**0.039**
Smoke	13 (6.0)	4 (5.8)	7 (9.1)	2 (2.9)	0.323
Alcohol	101 (47.0)	28 (40.6)	39 (50.6)	34 (49.3)	0.45
Spicy food	123 (57.2)	41 (59.4)	48 (62.3)	34 (49.3)	0.261
Exercise	44 (20.5)	22 (31.9)	18 (23.4)	4 (5.8)	**< 0.001**
Sun exposure	92 (42.8)	32 (46.4)	34 (44.2)	26 (37.7)	0.571
Applying cosmetics	2 (0.9)	0 (0.0)	2 (2.6)	0 (0.0)	0.331
Using or changing skincare products	14 (6.5)	6 (8.7)	8 (10.4)	0 (0.0)	**0.008**
Emotional changes	83 (38.6)	36 (52.2)	31 (40.3)	16 (23.2)	**0.002**

*Note:* Statistically significant *p* values are in bold.

### 3.3 Comorbidities, Psychological States, and Therapeutic Tendencies

Notably, 26.0% of patients reported concurrent skin conditions alongside rosacea. Acne vulgaris (9.8%) and seborrheic dermatitis (8.8%) were the most frequently reported conditions. Patients aged ≥ 45 years were more likely to have both eczema and rosacea (*p* = 0.006). Furthermore, 14% of patients reported systemic diseases, with a significant variance in distribution among the three age groups. Among these, cardiovascular disease was the most prevalent systemic ailment (5.1%), particularly in patients aged 45 years and older (*p* = 0.005), whereas neuropsychiatric disease was most frequently reported in patients aged ≤ 30 years (*p* = 0.05). Additionally, patients reported comorbid endocrine (2.8%), digestive (3.7%), genitourinary (1.9%), and respiratory (1.4%) disorders.

The HADS results revealed that among the 215 male patients, 27.9% experienced anxiety, and 26.0% experienced depression, with the majority of patients classified as having mild anxiety (17.2%) or mild depression (18.1%). There were no significant differences in the prevalence of anxiety or depression across the different age groups. However, the statistical analysis indicated group disparities in patients with moderate anxiety (*p* = 0.006) and moderate depression (*p* = 0.047), with the highest proportion observed in patients aged ≤ 30 years. Approximately 82.8% of the patients had previously sought treatment for rosacea, with a statistically significant difference between the age groups (*p* = 0.031), with the highest percentage in group one (92.8%). Notably, all included patients had an average of 1.66 ± 0.18 visits to our hospital, with most patients opting for systemic medications (66.0%) and physical modalities (52.1%). In comparison, 39.5% of the participants used topical medications, with no discernible differences between the age groups (Table 3).

**TABLE 3:** Comorbidities, psychological state, and therapeutic tendency of the different age groups [*n* (%), mean ± SD].

Variable	Total (*n* = 215)	≤ 30 (*n* = 69)	31–44 (*n* = 77)	≥ 45 (*n* = 69)	*p*
**Other skin diseases**	56 (26.0)	19 (27.5)	18 (23.4)	19 (27.5)	0.822
Acne vulgaris	21 (9.8)	9 (13.0)	7 (9.1)	5 (7.2)	0.550
Seborrhoeic dermatitis	19 (8.8)	5 (7.2)	9 (11.7)	5 (7.2)	0.524
Contact dermatitis	7 (3.3)	3 (4.3)	1 (1.3)	3 (4.3)	0.453
Eczema	5 (2.3)	0 (0.0)	0 (0.0)	5 (7.2)	**0.006**
Atopic dermatitis	1 (0.5)	1 (1.4)	0 (0.0)	0 (0.0)	0.642
Vitiligo	1 (0.5)	1 (1.4)	0 (0.0)	0 (0.0)	0.642
Photosensitivity	2 (0.9)	0 (0.0)	2 (2.6)	0 (0.0)	0.331
Psoriasis	2 (0.9)	0 (0.0)	1 (1.3)	1 (1.4)	1.000
**Systemic diseases**	30 (14.0)	6 (8.8)	8 (10.5)	16 (22.5)	**0.036**
Endocrine metabolic system	6 (2.8)	1 (1.4)	1 (1.3)	4 (5.8)	0.325
Cardiovascular system	11 (5.1)	0 (0.0)	3 (3.9)	8 (11.6)	**0.005**
Digestive system	8 (3.7)	1 (1.4)	4 (5.2)	3 (4.3)	0.543
Neuropsychiatric system	7 (3.3)	5 (7.2)	2 (2.6)	0 (0.0)	**0.050**
Genitourinary system	4 (1.9)	1 (1.4)	0 (0.0)	3 (4.3)	0.119
Respiratory system	3 (1.4)	0 (0.0)	0 (0.0)	3 (4.3)	0.064
**HADS (anxiety)**					
None	155 (72.1)	46 (66.7)	54 (70.1)	55 (79.7)	0.210
Mild	37 (17.2)	11 (15.9)	15 (19.5)	11 (15.9)	0.829
Moderate	16 (7.4)	11 (15.9)	3 (3.9)	2 (2.9)	**0.006**
Severe	7 (3.3)	1 (1.4)	5 (6.5)	1 (1.4)	0.224
**HADS (depression)**					
None	159 (74.0)	54 (78.3)	57 (74.0)	48 (69.6)	0.499
Mild	39 (18.1)	8 (11.6)	16 (20.8)	15 (21.7)	0.248
Moderate	11 (5.1)	7 (10.1)	1 (1.3)	3 (4.3)	**0.047**
Severe	6 (2.8)	0 (0.0)	3 (3.9)	3 (4.3)	0.253
**Therapeutic inclinations**					
Any previous treatment	178 (82.8)	64 (92.8)	60 (77.9)	54 (78.3)	**0.031**
Number of visits to our hospital	1.66 ± 1.37	1.86 ± 1.68	1.66 ± 1.32	1.48 ± 1.04	0.265
Use of topical medicines	85 (39.5)	29 (42.0)	28 (36.4)	28 (40.6)	0.775
Use of systemic medicines	142 (66.0)	42 (60.9)	54 (70.1)	46 (66.7)	0.489
Use of physical modalities	112 (52.1)	32 (46.4)	48 (62.3)	32 (46.4)	0.081

*Note:* Statistically significant *p* values are in bold. Data are *n* (%), mean ± standard deviation (SD).

### 3.4 Risk Factors for Early Onset of Male Rosacea

**FIGURE 1:**
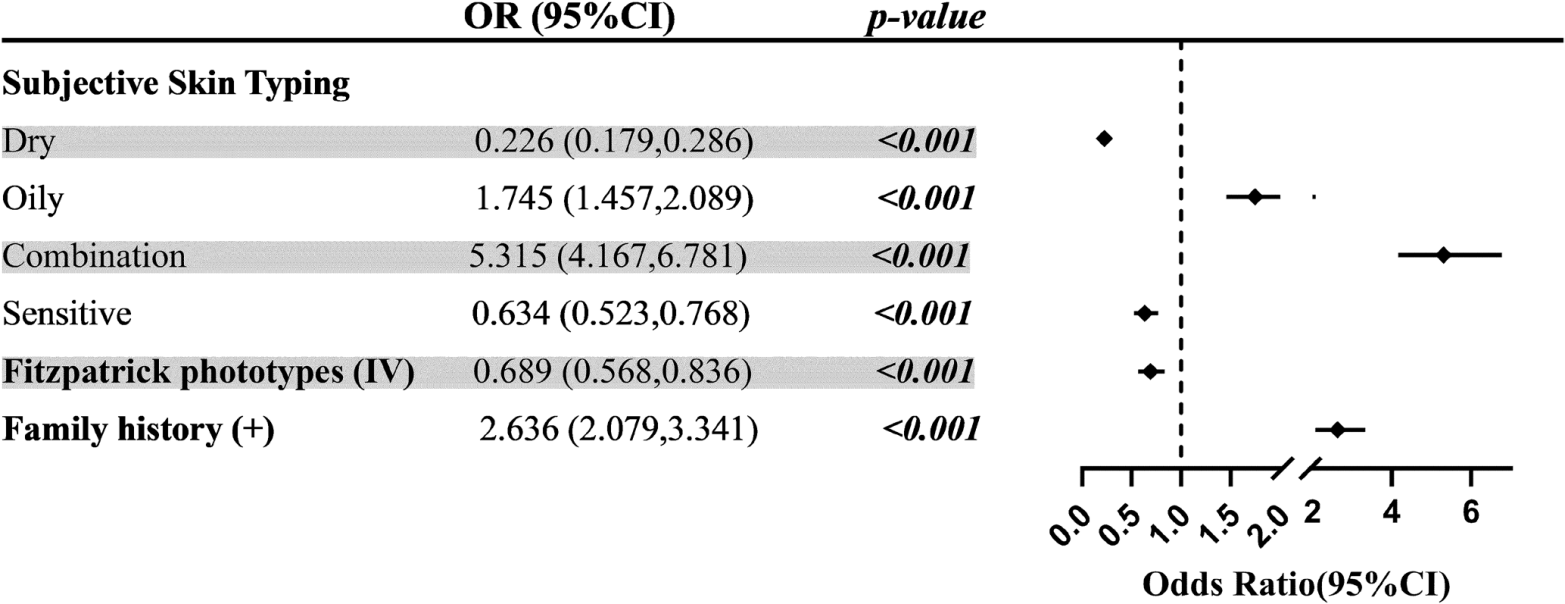
Risk factors for early‐onset rosacea in males according to multivariate analysis.

Based on the reported age of onset, patients were divided into two groups: Those with an onset age ≤ 30 years (*n* = 107, 49.8%) and those with an onset age > 30 years (*n* = 108, 51.2%). Single‐factor analysis was conducted on selected characteristics from the patients' baseline information, and the results are shown in Table 4. Subjective skin typing (*p* < 0.001), Fitzpatrick phototyping (*p* = 0.003), and family history (*p* = 0.026) were found to be associated with the age of onset in male patients with rosacea. The results of the multifactorial regression are presented in Figure 1. After adjusting for patient age, compared to male patients with normal skin, male patients with dry or sensitive skin tended to have a later onset, and patients with Fitzpatrick phototype IV were more likely to have a later onset than patients with type II or type III skin. Oily skin, combination skin, and a positive family history are risk factors for male patients with rosacea to develop this condition before the age of thirty.

**TABLE 4:** Baseline characteristics distribution between patients of different onset ages [*n* (%), mean ± SD].

Variable	Total (*n* = 215)	Age of onset ≤ 30 (*n* = 107)	Age of onset > 30 (*n* = 108)	*p*
Age	38.59 ± 13.13	29.60 ± 8.79	47.43 ± 10.48	/
Age of onset	32.68 ± 13.21	21.96 ± 5.14	43.35 ± 9.74	/
Subjective skin typing				
Dry	33 (15.3)	8 (7.5)	25 (23.1)	**< 0.001**
Normal	35 (16.3)	14 (13.1)	21 (19.4)	
Oily	68 (31.6)	42 (39.3)	26 (24.1)	
Combination	29 (13.5)	23 (21.5)	6 (5.6)	
Sensitive	50 (23.3)	20 (18.7)	30 (27.8)	
Fitzpatrick phototypes				
II	6 (2.8)	6 (5.6)	0 (0.0)	**0.003**
III	185 (86.0)	94 (87.9)	91 (84.3)	
IV	24 (11.2)	7 (6.5)	17 (15.7)	
Family history	22 (10.2)	16 (15.0)	6 (5.6)	**0.026**
Other skin diseases	56 (26.0)	31 (29.0)	25 (23.1)	0.354
Systemic diseases	30 (14.0)	11 (10.3)	19 (15.1)	0.088

*Note:* Statistically significant *p* values are in bold. Data are n(%), mean ± standard deviation (SD).

## 4 Discussion

Retrospective analyses of 215 male patients revealed that male patients with rosacea exhibit unique characteristics, with clinical features varying among different age groups. The mean age of the male patients was 38.59 ± 13.13 years. Comparatively, studies in Germany and Russia reported a mean age range of 42–50 years for patients with rosacea [11, 21]. A retrospective Korean study indicated a mean age of 48.8 ± 14.8 years for male patients [12]. Notably, the mean age of our male patients was younger, which aligns more closely with findings from studies conducted in China [17, 22]. The mean age at onset was 32.68 ± 13.21 years, suggesting that rosacea typically manifests after the age of 30 years, with a predilection for middle‐aged individuals among men. In terms of subtype distribution, ETR was the most prevalent subtype of rosacea among males across all age brackets, mirroring the subtype distribution observed in mixed sexes and females [12, 17, 22]. However, in males, the proportion of PhRs ranked second only to that of ETRs. This finding contrasts with the subtype distribution among female patients with rosacea, revealing a comparatively greater prevalence of PhRs among male patients [17]. This observation is in line with the outcomes of a comprehensive meta‐analysis focusing on the subtypes of rosacea [14].

Family history was documented in 10.2% of male patients, a figure akin to the 10.7% reported in the Korean study but notably lower than figures observed in studies conducted in Colombia, Germany, and Russia [21, 23]. Among the 840 female patients with rosacea included in our previous analysis, 6.7% reported a family history [22], with these patients attending the same hospital as the male patients in this study. Given the proximity in both geographical and temporal aspects, we postulate that male patients with rosacea exhibit a more pronounced family history than their female counterparts, possibly indicating a greater genetic predisposition among males. Furthermore, a notable disparity in family history was observed across different age groups, with patients aged ≤ 30 years reporting a greater prevalence of family history. This observation suggested a potential correlation between a clear family history of rosacea and an earlier age of onset among male patients, which was also confirmed by the risk factor analysis conducted in this study. In addition to a positive family history, both univariate analysis and multiple regression analysis revealed that patients' skin conditions, including skin type and Fitzpatrick phototype, have an impact on the age of disease onset. In fact, skin type and phototype are also genetically related, so our findings confirm that rosacea is a genetically related disease.

Given the prevalence of mixed subtypes of patients, we used the recommended approach of analyzing clinical features based on phenotypes [19, 24]. Persistent facial erythema and telangiectasia are the most common phenotypes in male patients. Although their distribution does not differ across different age groups, their severity increases with age, similar to our previous observations in female patients [22]. However, compared with females, male patients exhibited a significantly greater prevalence of phymatous changes (42.3% vs. 6.4%). Furthermore, both the distribution and severity of phymatous changes vary across different age groups, with males aged ≥ 45 showing the highest distribution and severity. This may be attributed to the longer disease duration in this age group and hormonal changes associated with aging, leading to skin thickening and increased visibility of phymatous changes. Patients aged ≥ 45 years reported the highest proportion of pruritus, suggesting a potential association between phymatous manifestations and itching. Patients aged ≤ 30 years tend to exhibit more involvement of the nasal area, whereas those aged ≥ 45 years tend to show more involvement of the cheeks. One study indicated that male sex and family history might be risk factors for nasal involvement [15]. Given that we reported more family history among patients aged ≤ 30 years, this could explain the distribution of skin lesions in our study. Furthermore, male patients with rosacea tend to exhibit more nasal involvement than female patients, which aligns with our research findings [17, 22]. Previous studies have also reported that a significant number of patients experience worsening of rosacea due to changes in temperature and sun exposure [12]. Interestingly, we observed a greater proportion of patients reporting exacerbation due to spicy food, possibly because our hospital is located in a province in China where chili consumption is prevalent in daily life for most patients. Furthermore, compared to studies focusing on mixed‐gender or female patients, our male patients reported a greater proportion of exacerbations due to alcohol and a lower proportion due to emotional changes [17, 25]. Factors such as exercise and emotional changes appear to have greater impacts on younger patients. This may be attributed to the decreased SIRT7 associated with skin aging in older patients, leading to reduced skin immunoreactivity.

According to mixed‐gender studies [12], seborrheic dermatitis and acne are the most commonly associated skin conditions in males. Eczema, due to its age‐related onset, tends to be more commonly associated with older patients. Previous research has established an association between rosacea and systemic diseases. Our study indicated that rosacea is concomitant with endocrine, digestive, respiratory, cardiovascular, neurological, psychiatric, and genitourinary system diseases, consistent with previous research [22, 25]. Older patients are more prone to systemic comorbidities, particularly cardiovascular diseases, emphasizing the need for dermatologists to pay closer attention to the overall health of patients, especially those aged ≥ 45 years. Furthermore, younger patients with rosacea appear to be more susceptible to psychiatric comorbidities, highlighting the importance of addressing mental health in the diagnosis and treatment of rosacea. As a disfiguring condition that may impact patients' mental health, we assessed their psychological state. In comparison to our previous research focused on female patients, the male cohort showed lower proportions of anxiety and depression [22]. From a sociopsychological perspective, the psychological burden of skin disease may be less severe for males than for females. However, a greater proportion of patients aged ≤ 30 years had moderate anxiety/depression. Overall, among males, younger patients may be more concerned about skin conditions. This finding aligns with our findings that a greater proportion of younger male patients sought alternative treatments before visiting our hospital, indicating a greater willingness among younger patients to improve their skin condition, although the difference was not statistically significant. Patients aged ≤ 30 years also had the highest average number of visits to our dermatology department.

Our research provides a detailed description of the clinical characteristics of male patients with rosacea, compares different age groups and discusses potential factors influencing the age of onset. To our knowledge, this is the first retrospective study specifically focused on male patients with rosacea. We also compared certain characteristics of male patients with our team's previously published research on female patients, demonstrating the coherence and consistency of our research. The strength of this article lies in the fact that these data were collected and evaluated by professional dermatologists in outpatient settings, with almost no missing information. However, the article has limitations. In terms of comorbidities, we relied on patient self‐reports, which may introduce biases due to inadequate patient disease awareness. Additionally, as a large tertiary hospital, our patients may exhibit more severe symptoms, which should be considered when evaluating the overall condition of the disease in the population.

## 5 Conclusion

A retrospective analysis of 215 male patients revealed that the predominant subtypes of rosacea were ETR and PhR, with persistent erythema and telangiectasia being the main symptoms. There was a high prevalence of phymatous changes and nasal involvement. Variations were observed across different age groups in terms of family history, rosacea features, severity, triggering factors, and comorbidities, and male rosacea patients demonstrated unique clinical characteristics compared to their female counterparts. The age of onset is influenced by subjective skin typing, Fitzpatrick phototype, and family history, with genetic factors possibly being an important consideration in the onset of male rosacea.

## Author Contributions

Acquisition of data: Yuwei Huang and Xu Liu; Analysis and interpretation of data: All authors; Drafting of the manuscript: Yuwei Huang; Critical revision of the manuscript for important intellectual content: Siliang Chen and Dan Du; Obtained funding: Xian Jiang; Study supervision: Xian Jiang.

## Ethics Statement

This study was approved by the Medical Ethics Committee of the West China Hospital of Sichuan University (Clinical Trials.gov ID: 2024–237). The work described has been carried out under The Code of Ethics of the World Medical Association (Declaration of Helsinki).

## Conflicts of Interest

The authors declare no conflicts of interest.

## Data Availability

The data that support the findings of this study are available on request from the corresponding author. The data are not publicly available due to privacy or ethical restrictions.
